# Compositional profile of α/β-hydrolase fold proteins in mangrove soil metagenomes: prevalence of epoxide hydrolases and haloalkane dehalogenases in oil-contaminated sites

**DOI:** 10.1111/1751-7915.12157

**Published:** 2014-08-29

**Authors:** Diego Javier Jiménez, Francisco Dini-Andreote, Júlia Ronzella Ottoni, Valéria Maia de Oliveira, Jan Dirk van Elsas, Fernando Dini Andreote

**Affiliations:** 1 Department of Microbial Ecology, Centre for Ecological and Evolutionary Studies, University of GroningenGroningen, 9747AG, The Netherlands; 2 Microbial Resources Division, Research Center for Chemistry, Biology and Agriculture, University of CampinasCampinas, SP, 6171, Brazil; 3 Department of Soil Science, ‘Luiz de Queiroz’ College of Agriculture, University of São PauloPiracicaba, SP, CEP, 13418-900, Brazil

## Abstract

The occurrence of genes encoding biotechnologically relevant α/β-hydrolases in mangrove soil microbial communities was assessed using data obtained by whole-metagenome sequencing of four mangroves areas, denoted BrMgv01 to BrMgv04, in São Paulo, Brazil. The sequences (215 Mb in total) were filtered based on local amino acid alignments against the Lipase Engineering Database. In total, 5923 unassembled sequences were affiliated with 30 different α/β-hydrolase fold superfamilies. The most abundant predicted proteins encompassed cytosolic hydrolases (abH08; ∼ 23%), microsomal hydrolases (abH09; ∼ 12%) and *Moraxella* lipase-like proteins (abH04 and abH01; < 5%). Detailed analysis of the genes predicted to encode proteins of the abH08 superfamily revealed a high proportion related to epoxide hydrolases and haloalkane dehalogenases in polluted mangroves BrMgv01-02-03. This suggested selection and putative involvement in local degradation/detoxification of the pollutants. Seven sequences that were annotated as genes for putative epoxide hydrolases and five for putative haloalkane dehalogenases were found in a fosmid library generated from BrMgv02 DNA. The latter enzymes were predicted to belong to *Actinobacteria*, *Deinococcus-Thermus*, *Planctomycetes* and *Proteobacteria*. Our integrated approach thus identified 12 genes (complete and/or partial) that may encode hitherto undescribed enzymes. The low amino acid identity (< 60%) with already-described genes opens perspectives for both production in an expression host and genetic screening of metagenomes.

## Introduction

Mangroves harbour diverse microbial communities which play critical roles in the functioning and maintenance of these sensitive and complex systems (Kathiresan and Bingham, 2001; Sahoo and Dhal, 2006). Given the peculiar factors that drive these systems (salt, anaerobic/aerobic shifts), mangroves offer rich sources of genes for new biotechnological products/enzymes, such as lipases (Couto *et al*., 2010), cellulases (Thompson *et al*., 2013) and laccases (Ye *et al*., 2010). Mangrove soils have already been explored for microbial diversity using a wide range of culture-dependent and culture-independent methods (Dias *et al*., 2009; dos Santos *et al*., 2011). In particular, the modern metagenomics-based tools, i.e. high-throughput sequencing of environmental DNA followed by a directed search for target genes, allow a ready access to the metabolic potential of mangrove microbial communities (Andreote *et al*., 2012). Moreover, genes for important enzymes can be further custom-synthesized and codon-optimized, after which heterologous expression may be achievable in a suitable host, an approach that has been coined ‘synthetic metagenomics’ (Chistoserdova, 2010). To access whole operons, metagenomic libraries can be constructed in large-insert vectors and screened either by functional or genetic approaches. Importantly, functional screening analysis does not depend on prior sequence information to detect the target proteins, provided these become available and active in the novel host. Recently, it was shown that particular sequence/activity incoherencies in databases can be solved using expression detection (Fernández-Arrojo *et al*., 2010; Jiménez *et al*., 2012a). However, proper testing requires that the expression conditions in the heterologous host are adequate (Ekkers *et al*., 2012).

Current classification of metagenomic sequencing data relies strongly on local alignments (e.g. using blast) against public databases (e.g. NCBI, SEED and KEGG) (Montaña *et al*., 2012). However, completely novel biotechnologically relevant proteins cannot be easily discovered using such approach. Metagenome datasets can be assessed by the use of more specific databases, such as for example the CAZy (Carbohydrate-Active Enzyme) (Cantarel *et al*., 2008), PeroxiBase (Fawal *et al*., 2013), Lipase/Laccase Engineering (Fischer and Pleiss, 2003; Sirim *et al*., 2011), ESTHER (Lenfant *et al*., 2013), 3DM (Kourist *et al*., 2010), Epoxide Hydrolases and/or Haloalkane Dehalogenases (Barth *et al*., 2004). Alternatively, catalytic or structurally conserved domains can be detected using hidden Markov models (HMM). However, most HMM are designed based on protein sequences retrieved from databases and so the true novelty is still questionable.

The α/β-hydrolase fold enzymes, present in the Lipase Engineering Database (LED), constitute a protein family with diverse catalytic and non-catalytic functions. The α/β-hydrolase fold proteins consist of eight β-strands connected by α-helices. These enzymes are characterized by a common catalytic triad formed by a catalytic nucleophile (serine, aspartate or cysteine), a histidine and an acidic residue (aspartate or glutamate). These residues occur on conserved locations in loops and the α/β-hydrolase fold brings them together to form the active site (Lenfant *et al*., 2013). These proteins encompass several key enzymes for biocatalytic applications, e.g. lipases, esterases, epoxide hydrolases (EHs), C–C breaking enzymes, dehalogenases and hydroxynitrile lyases (Holmquist, 2000).

Interestingly, EHs (Enzyme Commission number-EC 3.3.2.9) from microbial sources have been recently recognized as a versatile group of enzymes that are important for the synthesis of enantiopure oxides and vicinal diols (intermediates in the organic synthesis of chiral pharmaceutical compounds, drugs and agrochemicals) (Lee and Shuler, 2007; Choi, 2009; Sareen and Kumar, 2011). Such EHs are involved in the degradation of several hydrocarbons including 1,3-dihalo-2-propanol, epichlorohydrin, 9,10-epoxy fatty acids, trans-2,3-epoxysuccinate and 2,3- chlorostyrene oxides (van der Werf *et al*., 1998; Fretland and Omiecinski, 2000). The presence of EHs has been reported in bacteria recovered from gasoline and oil-contaminated marine sediments (Kwon *et al*., 2007; 2010; Woo *et al*., 2007; 2013). Moreover, haloalkane dehalogenases (HDs) (EC 3.8.1.5) have attracted considerable attention due to their unique catalytic mechanism, broad substrate specificity, stability, enantioselectivity and catalytic efficiency (Koudelakova *et al*., 2013). The HDs catalyse the cleavage of carbon–halogen bonds, which is a key step in the aerobic mineralization of many halogenated pollutants, such as oil compounds (Janssen *et al*., 2005). Previous studies indicated HDs to be important for the preparation of optically pure building blocks for organic synthesis, recycling of by-products from chemical processes, decontamination of chemical warfare agents and for bio-sensing of environmental pollutants and protein tagging for cell imaging and protein analysis (Koudelakova *et al*., 2013).

The current study aimed at bioprospection of shotgun sequence datasets generated from four mangrove soils for α/β-hydrolase fold proteins by using specific LED. In addition, a metagenomic fosmid library constructed from one oil-impacted mangrove site (BrMgv02) was used for sequence-based screening. The prevalence [relative abundance (RA)] of EHs and HDs was addressed, with special reference to the oil contamination, biodegradation and future potential industrial application.

## Results and discussion

**Fig 1:**
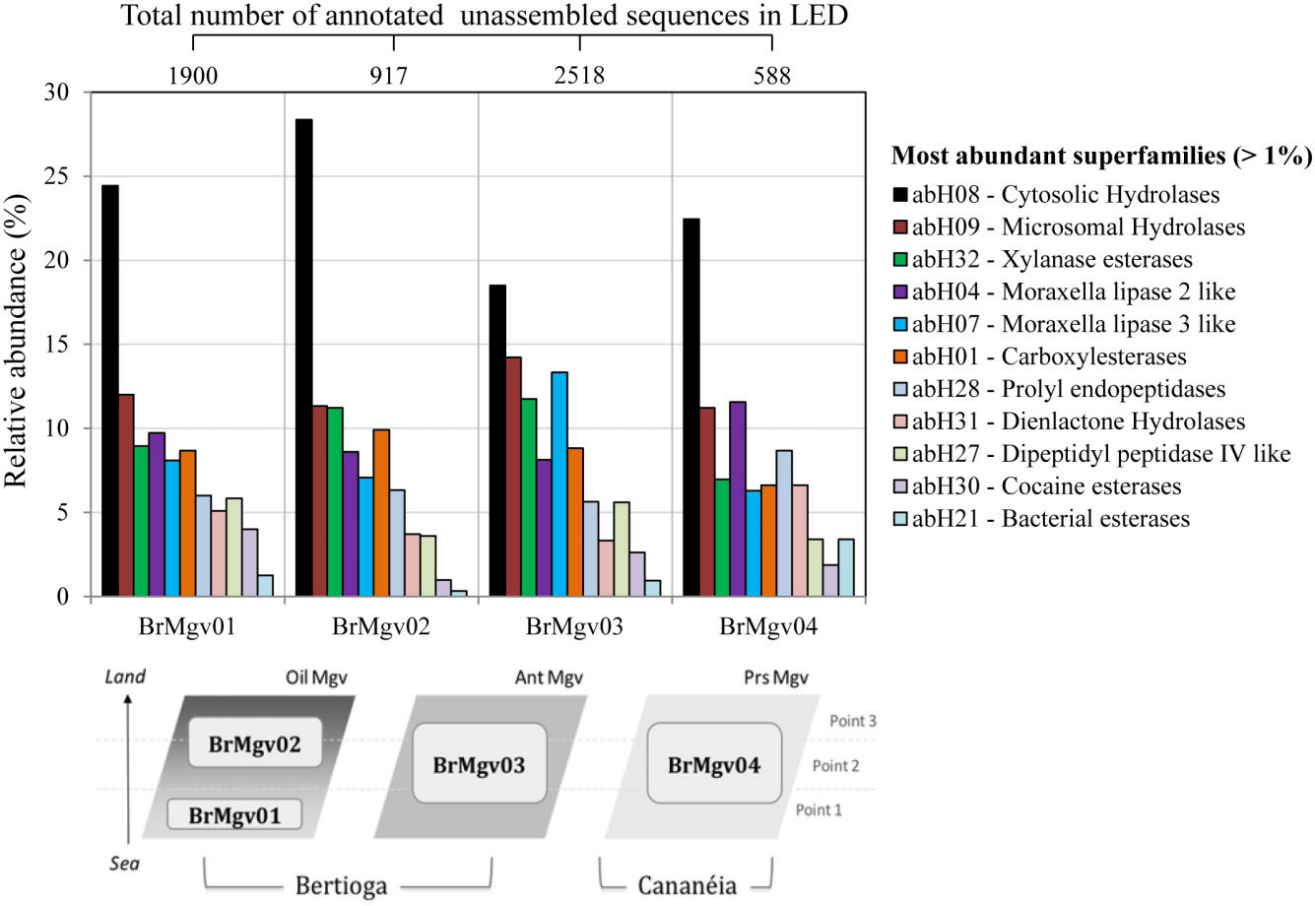
Relative abundance (%) of the most abundant α/β-hydrolase fold protein superfamilies in the four mangrove metagenomes (BrMgv01 to BrMgv04). Oil Mgv: oil-polluted mangrove site; Ant Mgv: anthropogenic polluted site; Prs: pristine mangrove site. The database was downloaded from the LED ftp website – http://www.led.uni-stuttgart.de/ – release 3.0, last update on 10 December 2009. Normalization was performed using the total number of annotated sequences (in LED) in each dataset.

In this study, an analysis of the composition and diversity of metagenomic sequences encoding α/β-hydrolase fold proteins in four distinct mangrove soils was performed. Briefly, mangrove soil samples were collected in July 2008 in three distinct mangroves in the state of São Paulo (Brazil). Samples were divided in four groups: BrMgv01 and BrMgv02 (23°53′49″S; 46°12′28″W – Bertioga city) are two sites in the same mangrove separated by a small stream. This area has been affected by oil contamination (petroleum). Sample BrMgv03 (23°54′06″S; 45°15′03″W – Bertioga city) was taken from a site adjacent to BrMgv01-02 that had not been affected by oil, but by household waste. Finally, BrMgv04 (25°05′02″S; 47°57′42″W – Cananéia city) represents a sample from a pristine mangrove (Fig. 1) as detailed in Andreote and colleagues (2012). In total, 1.8 g (six samples per site) of soil from each area was subjected to total genomic DNA extraction, after which the DNAs were subjected to shotgun sequencing using the 454 GS-FLX titanium technology (Indianapolis, IN, USA). The sequences obtained (905 521 unassembled sequences with an average length of 236 bp) were sorted and trimmed based on length and quality, using an in-house python script (Jiménez *et al*., 2012b). The total numbers of trimmed sequences obtained for each mangrove area were 249 993 for BrMgv01 (average read length ∼ 235 bp), 231 233 for BrMgv02 (∼ 238 bp), 214 921 for BrMgv03 (∼ 248 bp) and 217 605 for BrMgv04 (∼ 223 bp). These sequences were uploaded to the metagenomic RAST (MG-RAST) server and made publically accessible under the project codes 4451033.3, 4451034.3, 4451035.3 and 4451036.3 for mangroves BrMgv01, BrMgv02, BrMgv03, and BrMgv04 respectively. As a complement to a previous study (Andreote *et al*., 2012), we performed BLASTX against LED (Fischer and Pleiss, 2003) using a cut-off e-value of 1e-5, as in other studies in which moderately rigid criteria were used to find genetic novelty and to evaluate functional and taxonomic profiles (Jung *et al*., 2011; Jiménez *et al*., 2012b; Mendes *et al*., 2014). It is important to note that BLASTX has been successfully used against LED in other studies (Kim *et al*., 2009; Damon *et al*., 2012). However, we are aware of the fact that these parameter settings may result in spurious hits and thus data need to be carefully re-examined. To address this critical issue, we performed manual annotation in the best hits. With this strategy, the information retrieved was compared across the four datasets. Thus, totals of 1900 (0.8% RA), 917 (0.4% RA), 2518 (1.1% RA) and 588 (0.2% RA) unassembled sequences were found to match 30 different α/β-hydrolase superfamilies, for mangrove samples BrMgv01, BrMgv02, BrMgv03 and BrMgv04 respectively (Fig. 1). Collectively, these sequences matched 22 superfamilies in the class GGGX. In the LED, proteins were assigned to the classes GX, GGGX and Y, in accordance with their amino acid sequences and the structures of the oxy anion holes (Pleiss *et al*., 2000). The oxy anion hole helps to stabilize the negatively charged transition state that occurs during enzymatic hydrolysis (Nardini and Dijkstra, 1999).

The most abundant superfamilies across all metagenomes were annotated as cytosolic hydrolases (abH08) (∼ 23% RA) and microsomal hydrolases (abH09) (∼ 12% RA) (except in BrMgv04), followed by *Moraxella* lipase 2-like sequences (abH04) (9.7% RA in BrMgv01 and 11.5% RA in BrMgv04), xylanase esterases (abH32) (11.7% RA in BrMgv02) and *Moraxella* lipase 3-like (abH07) (13.3% RA in BrMgv03) (Fig. 1). Superfamily abH08 consists of 15 homologous families that include 3188 protein entries in LED. Into the abH08 superfamily, we can found a large group of bacterial EHs, non-heme peroxidases and HDs. In addition, superfamily abH09 contains three families, namely microsomal EHs, BioH protein like (biotin biosynthesis) and proline iminopeptidases (Fischer and Pleiss, 2003). Furthermore, proline iminopeptidases, propyl endopeptidases and dipeptidyl peptidases make part of the bacterial proteolytic system, which has been reported as being key for bacterial nitrogen utilization (nitrogen from amino acids), especially under nitrogen-limiting conditions (Kunji *et al*., 1996; Li *et al*., 2010). Nitrogen limitation may reign in anoxic soil mangroves, in which denitrification leading to gaseous nitrogen effluxes is rampant (Fernandes *et al*., 2012).

**Fig 2:**
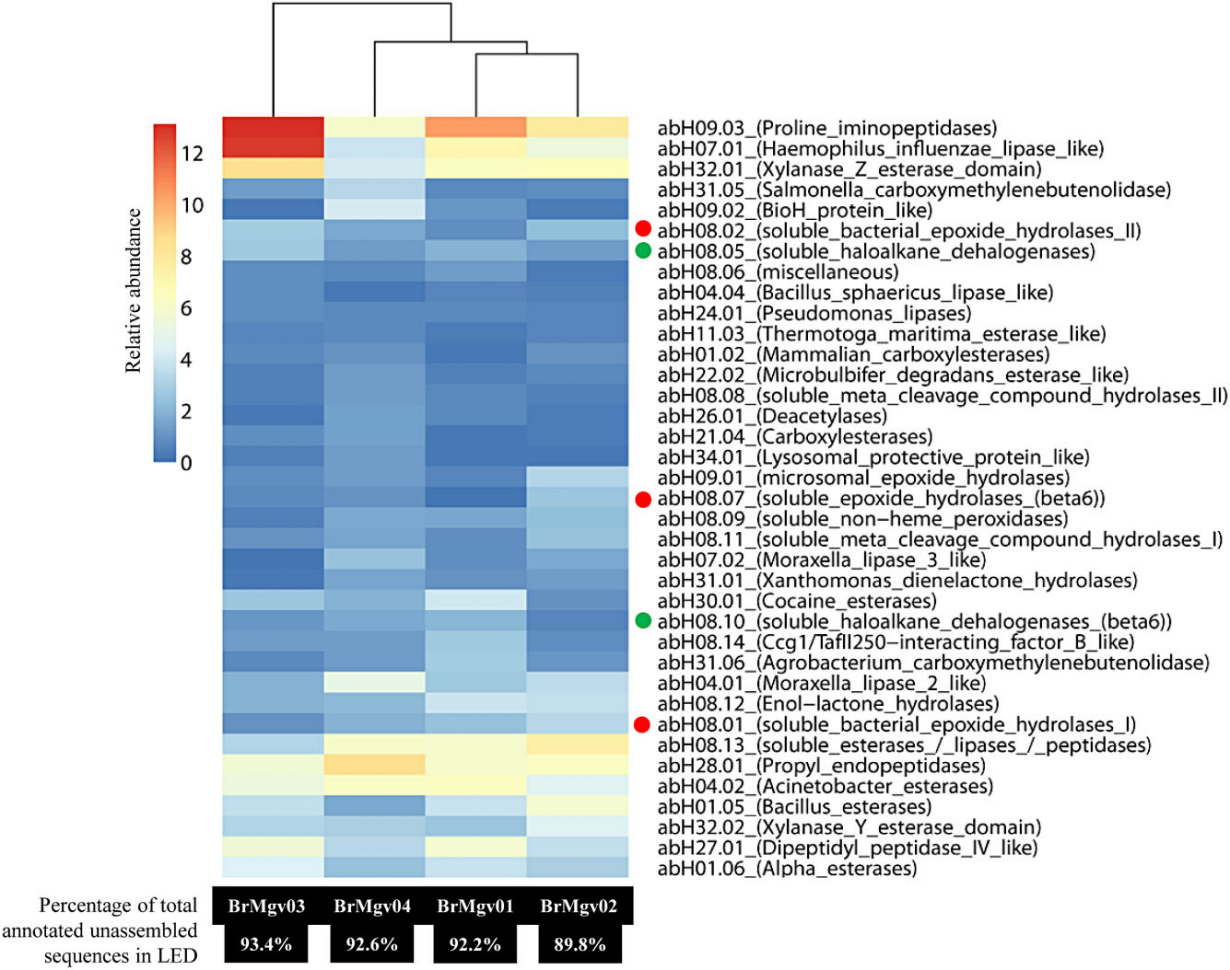
Relative abundance (%) of the most abundant α/β-hydrolase fold protein families in four mangrove metagenomes (BrMgv01-BrMgv04). Hierarchical dendrogram and heat-mapping were performed using cluster V3.0 (Eisen *et al*., 1998) software. Red and green dots represent the epoxide hydrolase (EHs) and haloalkane dehalogenase (HDs) families respectively.

We then assessed the distribution of the unassembled sequences over the predicted protein families per mangrove sample. The prevalences of proline iminopeptidases (abH09.03), *Haemophilus influenzae* lipase-like (abH07.01), xylanase Z esterase domain (abH32.01) and dipeptidyl peptidases (abH27.01) were highest in BrMgv01 (10.3%, 7.2%, 6.4% and 5.8% RA respectively) and BrMgv03 (13.1%, 12.7%, 8.5% and 5.5% RA respectively). The families encompassing *Acinetobacter* esterases (abH04.02) and propyl endopeptidases (abH28.01) showed highest prevalences (6.3 and 8.6% RA respectively) in the unpolluted mangrove site (BrMgv04). Conversely, EHs, belonging to families abH08.01, abH09.01, abH08.07 and abH08.02, were abundant in BrMgv02 (3.3%, 3.1%, 2.6% and 2.3% RA respectively) compared with the unpolluted mangrove BrMgv04 (1.8%, 1.1%, 1.0% and 1.7% RA respectively) (Fig. 2). On another matter, genes encoding xylanases/esterases were very prevalent in all mangrove samples (between 4% and 8% RA), irrespective of pollution. This suggests that the conversion of hemicellulose is likely to occur in these environments, which are anaerobic most of the time (Benner *et al*., 1984; Ye *et al*., 2010).

**Fig 3:**
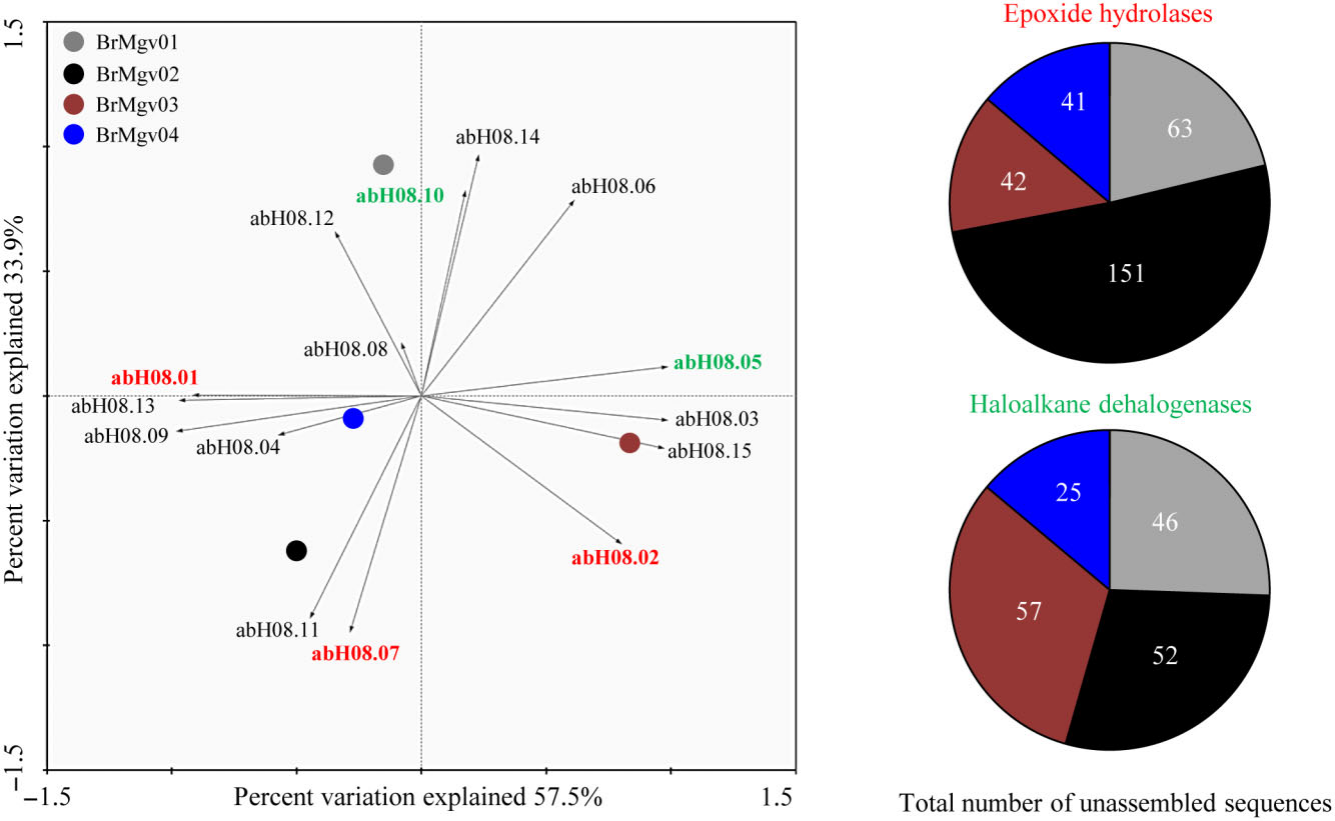
Left: principal component analysis (PCA) within the family abH08 using Canoco software v4.52 (Wageningen, the Netherlands). Right*:* Number of unassembled sequences annotated in the abH08.02 [epoxide hydrolases (EHs)] and abH08.05 [haloalkane dehalogenases (HDs)] families in BrMgv samples.

To shed light on the α/β-hydrolase fold protein superfamilies detected in our dataset, we focused on the protein superfamily that was most abundant across the board, i.e. abH08. Principal component analyses showed that EHs, families abH08.07 and abH08.02, were preferentially present in BrMgv02 and BrMgv03 respectively (Fig. 3). In terms of numbers of annotated sequences, the prevalences of these predicted enzymes (abH08.01, abH08.07 and abH08.02) were highest in BrMgv02 site (151 unassembled sequences – 30% RA based on the number of annotated sequences within abH08 family) comparatively to BrMgv04 (41 unassembled sequences – 17% RA). Regarding HDs (abH08.10 and abH08.05), highest numbers of annotated genes were observed in the polluted mangroves BrMgv02 (52 unassembled sequences – 11% RA) and BrMgv03 (57 unassembled sequences – 22% RA) (Fig. 3). Interestingly, van Loo and colleagues (2006) reported the screening of various genomic databases for the presence of EHs to find ways to express these proteins in different bacterial hosts. In addition, putative open reading frames for EHs and subsequent expression have been investigated in *Cupriavidus metallidurans* CH34 (Kumar *et al*., 2011). Clearly, novel EHs, provided they offer features such as enhanced activity, substrate specificity and/or stability in the face of chemicals, are useful in the production of a range of compounds (e.g. β3-adrenergic receptor agonists, anti-obesity and anti-inflammatory drugs, nematicides, anticancer agents, anti-fungal chemicals). In addition, they may also serve the detoxification of xenobiotics such as polyaromatic hydrocarbons and the production of enantiopure epoxides and vicinal diols from cheap racemic epoxides (Lee and Shuler, 2007).

In the light of the high prevalence of sequences related to EHs (family abH08.02) and HDs (family abH08.05) in highly oil-impacted mangrove BrMgv02 (73 and 36 unassembled sequences respectively), this specific dataset and the best BLAST hits (based on e-values) were further analysed. For the former unassembled sequences, we found some putative novel enzymes (based on relatively low amino acid identity: < 60%) that were mostly affiliated with similar proteins (amino acid identity between 50% and 73%) from *Streptomyces*, *Hyphomonas*, *Bradyrhizobium* and *Phenylobacterium*. For the abH08.05 family, HDs were affiliated to proteins from *Salinispora*, *Alcanivorax*, *Photobacterium*, *Moritella* and *Chloroflexus* (Table 1 – approach 1). It is important to stress that the taxonomic affiliation is only an indication of identity as it is based on function of the most similar or homologous proteins, and thus may not directly reflect the true microbial source. Thus a whole new suite of partial genes encoding novel enzymes within these two classes was unlocked. These are hypothesized to function best under the environmental conditions of the habitat, i.e. the local salt, oxic/anoxic conditions as well as the presence of hydrocarbons (Arulazhagan and Vasudevan, 2011; Arfi *et al*., 2013). Such enzymes might well suit industrial and environmental (pollutant removal) needs, as will be explored in future work. According to Andreote and colleagues (2012), the bacterial community at the BrMgv02 mangrove site were dominated by *Proteobacteria* belonging to the classes *Delta* < *Gamma* < *Alpha*. Moreover, *Alphaproteobacteria*, next to *Actinobacteria* were significantly raised compared with BrMgv04. Hence, we surmised that such bacterial groups were carriers of the biodegradative functions in the oil-polluted BrMgv02, which is consistent with findings in other estuarine ecosystems (Greer, 2010).

**Table 1:** Putative epoxide hydrolases (EHs) retrieved from the BrMgv02 dataset

Approach (Mangrove)	Best-hit protein (NCBI Accession No.)	Putative microbial source based on homology (Best hit protein)	Hit-length (aa)	Query-length (bp)	e-value	Amino acid identity (%)	ESTHER^c^
1 (BrMgv02)	Putative epoxide hydrolase (YP_761108)	*Hyphomonas neptunium*	320	345	7e-48	73.04	XE
	Epoxide hydrolase (NP_823281)	*Streptomyces avermitilis*	328	394	2e-28	48.46^a^	XE
	Epoxide hydrolase (YP_002203910)	*Streptomyces sviceus*	330	393	8e-28	47.69^a^	XE
	Epoxide hydrolase (NP_767754)	*Bradyrhizobium diazoefficiens*	330	345	7e-24	50^a^	XE
	Epoxide hydrolase (YP_002129963.1)	*Phenylobacterium zucineum*	321	347	4e-23	51^a^	XE
2 (BrMgv02)	Alpha/beta hydrolase fold protein (WP_008687080.1)	*Rhodopirellula sallentina*	319	850	1e-65	40^a^^b^	XE
	Epoxide hydrolase – like protein (YP_003705817.1)	*Frankia* sp.	398	351	4e-32	51^a^^b^	XE
	Alpha/beta hydrolase fold protein (YP_002544714.1)	*Truepera radiovictrix*	292	504	3e-38	69^b^	XE
	Alpha/beta hydrolase fold protein (YP_003336243.1)	*Streptosporangium roseum*	307	1459	3e-75	55^a^^b^	XE
	Epoxide hydrolase domain protein (WP_007534516.1)	*Rhizobium mesoamericanum*	199	345	3e-21	49^a^^b^	XE
	Epoxide hydrolase (WP_009025691)	*Bradyrhizobium* sp.	334	802	8e-74	46^a^^b^	XE
	Alpha/beta hydrolase (YP_727726.1)	*Rhodospirillum rubrum*	305	1039	4e-116	64^b^	XE

a Putative novel protein based on low amino acid identity (< 60%).

b Complete genes.

c Classification based on BLASTX against ESTHER database (Lenfant *et al*., 2013).

The table was constructed using two different approaches: (1) Whole metagenome sequencing – and BLASTX against the LED; (2) Fosmid library sequencing – and BLASTX against the NCBI using the MG-RAST pipeline.

XE, Block_X, Family Epoxide_hydrolase.

In a second stage, we studied selected sequences retrieved from a fosmid library constructed using the CopyControl Fosmid Library Production Kit – Epicentre which contains the pCC2FOS as a vector and *Escherichia coli* EPI 300 T1R Phage T1 resistant [F– mcrA Δ(*mrr-hsd*RMS-*mcr*BC) (StrR) φ80dl*ac*ZΔM15 Δl*ac*X74 *rec*A1 *end*A1 *ara*D139Δ(*ara, leu*)7697 *gal*U *gal*K λ– *rps*L *nup*G *trf*A *ton*A *dhfr*] as host cells. Environmental DNA extracted from the oil-impacted mangrove soil BrMgv02 was used to construct the metagenomic library, with insert sizes ranging from 25 to 40 kb. A total of 12 900 clones was obtained and further subjected to 454 pyrosequencing (GS FLX Titanium technology), yielding a total of 1 380 509 unassembled sequences with average length of 484 bp (approximately 624 Mb). The sequences were assembled into contigs using CLC Genomics Workbench (version 6.5.1; CLC Bio, Cambridge, MA, USA) (under default parameters) resulting in 118 882 sequences (average length 913 bp), totalling 108 Mb. The data were annotated using the MG-RAST pipeline (cut-off e-value of 1e-5) (Meyer *et al*., 2014). This allowed the detection of seven putative EHs in fosmids (fosmid library metagenomic data were deposited at MG-RAST under the ID No. 4555913.3). These were further compared and were found to be affiliated with EHs in genomes of *Rhodopirellula*, *Bradyrhizobium* (family abH08.02), *Truepera*, *Streptosporangium* (family abH08.01), *Rhizobium*, *Frankia* (family abH09.01) and *Rhodospirillum* (family abH08.07) (Table 1 – approach 2). Moreover, five HDs sequences were detected, and these matched those present in the genomes of *Anaeromyxobacter*, *Oceanicaulis* (family abH08.05), *Mycobacterium* (families abH08.05 and abH08.10) and *Aeromicrobium* (abH08.10). In some cases (five EHs and two HDs), the sequence identity with already described genes (database) was low (< 60%), suggesting that the sequences could represent putative new proteins. The hierarchical classification of these 22 (complete and/or partial) genes, based on the ESTHER database, was done (Tables 1 and 2). Importantly, the activity of such enzymes may not be detected in *E. coli* host cells, specially due to the different expression systems present in this species when compared with those of *Actinobacteria*, *Alphaproteobacteria*, *Deinococcus-Thermus* and *Planctomycetes* members (Gabor *et al*., 2004). On the other hand, the use of degenerate primers has been applied as a sequence-driven approach to identify enzymes directly from metagenomes. For example, Kotik and colleagues (2009) amplified fragments of EH genes using degenerate primers targeted to conserved motifs, followed by assembly by genome walking. These results highlight the importance of using both sequence and function-based approaches in metagenomic library screenings, thus circumventing problems inherent to either lack of heterologous expression efficiency or limited sequence information based on known protein sequences available at public databases. Our fosmid-based approach opens up the possibility of finding whole operons, start/stop codons and expression signals on the basis of the genetic information gathered in this study. The EHs and HDs encoding genes from the oil-impacted mangrove soils can thus be further expressed in appropriate expression vectors, for future practical use in industrial or biotechnological processes. Comparison of the EHs genes between the BrMgv02 metagenome and the fosmid library datasets revealed protein families (abH08.01, abH09.01, abH08.07 and abH08.02) to be coincident. Similar results were found with HDs, as the main protein families found within the BrMgv02 metagenome were also observed in the fosmid library dataset (abH08.05 and abH08.10). Thus, the metagenomic library represented to a considerable extent the diversity of EHs and HDs present in the analysed mangrove sample.

**Table 2:** Putative haloalkane dehalogenases (HDs) retrieved from the BrMgv02 dataset

Approach (Mangrove)	Best-hit protein (NCBI Accession No.)	Putative microbial source based on homology (Best hit protein)	Hit-length (aa)	Query-length (bp)	e-value	Amino acid identity (%)	ESTHER^c^
1 (BrMgv02)	Haloalkane dehalogenase (YP_001537995)	*Salinispora arenicola*	310	412	1e-29	65.8	X1
	Haloalkane dehalogenase (YP_694135)	*Alcanivorax borkumensis*	296	410	5e-29	63.5	X1
	Putative haloalkane dehalogenase (ZP_01221858)	*Photobacterium profundum*	303	242	1e-27	62.5	X1
	Putative haloalkane dehalogenase (ZP_01897865)	*Moritella* sp.	292	190	2e-25	75.8	X1
	Haloalkane dehalogenase (ZP_02986139)	*Chloroflexus* sp.	293	306	5e-19	45^a^	X1
2 (BrMgv02)	Haloalkane dehalogenase (YP_001377212.1)	*Anaeromyxobacter* sp.	304	840	8e-37	49^a^^b^	X1
	Haloalkane dehalogenase (WP_020727623.1)	*Mycobacterium marinum*	325	1752	1e-146	80^a^^b^	X2
	Haloalkane dehalogenase (WP_022699531.1)	*Oceanicaulis alexandrii*	301	249	1e-46	64^a^^b^	X1
	Haloalkane dehalogenase (YP_003336243.1)	*Mycobacterium vanbaalenii*	298	607	5e-81	61^a^^b^	X1
	Haloalkane dehalogenase (WP_007078410.1)	*Aeromicrobium marinum*	290	1241	3e-65	52^a^^b^	X1

a Putative novel protein based on low amino acid identity (< 60%).

b Complete genes.

c Classification based on BLASTX against ESTHER database (Lenfant *et al*., 2013).

The table was constructed using two different approaches: (1) Whole metagenome sequencing – and BLASTX against the LED; (2) Fosmid library sequencing – and BLASTX against the NCBI using the MG-RAST pipeline.

X1, Block_X, Family Haloalkane_dehalogenase-HLD1; X2, Block_X, Family Haloalkane_dehalogenase-HLD2.

Clearly, *Actinobacteria* and *Alphaproteobacteria* might serve as genetic sources for EHs bio-exploration. For instance, analysis of the genome of the actinobacterium *Mycobacterium tuberculosis* revealed an unusually large number of potential EHs, i.e. nine EHs genes occurred scattered on the genome (Johansson *et al*., 2005). Also, the presence of EHs in *Agrobacterium radiobacter* AD1 has been reported (Rink *et al*., 1997). These were further engineered towards an increasing activity for industrial purposes (Rui *et al*., 2005). Protein engineering proved to be an efficient method to tailor α/β-hydrolase fold enzymes towards a desired property. Moreover, enzymes with completely new catalytic activities have been generated, for instance the conversion of an esterase from *Pseudomonas fluorescens* into an EH (Jochens *et al*., 2009). The EHs, HDs and haloperoxidases have a typical lipase catalytic triad (G-X-S-X-G) and share approximately 25% of amino acid identity with lipases belonging to the family V (Arpigny and Jaeger, 1999; Tirawongsaroj *et al*., 2008). In the catalytic triad, the nucleophilic aspartate carries out an attack on the carbon atom of the epoxide ring, thus displacing the oxygen and producing a covalent intermediate compound (de Vries and Janssen, 2003). Soluble EHs have recently been found in an Andean forest soil metagenome, in this case affiliated to the bacterium *Streptomyces scabies* (Montaña *et al*., 2012). In addition, Procópio and colleagues (2013) reported the presence of five putative genes encoding EHs in the genome of *Dietzia cinnamea* (a common soil *Actinobacterium*). These studies are consistent with the notion that members of the *Actinobacteria* can produce EHs in the environment, which supports their potential use as bioremediation agents, for instance in oil-contaminated systems. Moreover, we also found HDs belonging to *Alcanivorax* and *Phenylobacterium*. These microorganisms are known as ‘hydrocarbonoclastic’ based on their capacity to degrade an exceptionally broad range of haloalkane hydrocarbons (Sabirova *et al*., 2006; dos Santos *et al*., 2011). The genus *Phenylobacterium* (a facultatively anaerobic bacterium) has a unique preference for phenyl moieties from heterocyclic compounds such as chloridazon, antipyrine and pyramidon (Oh and Roh, 2012). Conversely, *Alcanivorax* species has also been reported as a key bacterial group present in crude oil enrichments based on mangrove soils as the microbial source (Brito *et al*., 2006).

## Conclusions

Current bottlenecks in high-throughput metagenome analysis are mostly due to problems related to sequence annotation. This factor can drastically affect the interpretation of a given dataset, especially in the case of enzyme annotation (Hoff, 2009; Schnoes *et al*., 2009). In this sense, it becomes important to use specific and curated databases, which – in combination with manual annotation – can improve our capability of data mining. In this study, we make use of metagenomics datasets from mangrove soils to investigate the prevalence and diversity of genes for α/β-hydrolase fold related proteins, using a specific database. Sequences, predicted to belong to *Actinobacteria*, *Chloroflexi*, *Deinococcus-Thermus*, *Planctomycetes* and *Proteobacteria* EHs or HDs codifying genes are described and analysed. Moreover, the description of the EHs and HDs will be further explored in the context of the bioconversion of hydrocarbons in oil-contaminated environments. Our results might represent a first step towards the development of a totally synthetic metagenomics approach (synthesis-cloning-expression), to be broadly applied in mangrove tropical ecosystems. Finally, we conclude that the presence of hydrocarbons in mangrove soils has an effect on the abundance and diversity of α/β-hydrolase fold proteins, which were mostly heightened in EHs and HDs.
